# Supplementary material for: Cannabidiol Enhances Cabozantinib-Induced Apoptotic Cell Death via Phosphorylation of p53 Regulated by ER Stress in Hepatocellular Carcinoma
Source: Cancers (Basel). 2023 Aug 5;15(15):3987. doi: 10.3390/cancers15153987 (PMC10417827; doi:10.3390/cancers15153987)
Supplement: Supplementary file 1 [file cancers-15-03987-s001.zip › Supplementary figures.pdf]

Supplementary figures for:

Cannabidiol enhances cabozantinib-induced apoptotic cell death via phosphorylation of p53 regulated by ER stress in hepatocellular carcinoma

Authors: Youngsic Jeon[1,†], Taejung Kim[1,2,†], Hyukjoon Kwon[1], Jeong Kook Kim[3], Young-Tae Park[1], Jungyeob Ham[1,2,3,*] and Young-Joo Kim[1,*]

Author Affiliations:

[1] Natural Products Research Center, Korea Institute of Science and Technology, Gangneung, Republic of Korea

[2] Division of Bio-Medical Science & Technology, KIST School, University of Science and Technology, Seoul 02792, Republic of Korea,

[3] NeoCannBio Co., Ltd., Seoul, Republic of Korea

Supplementary Figures

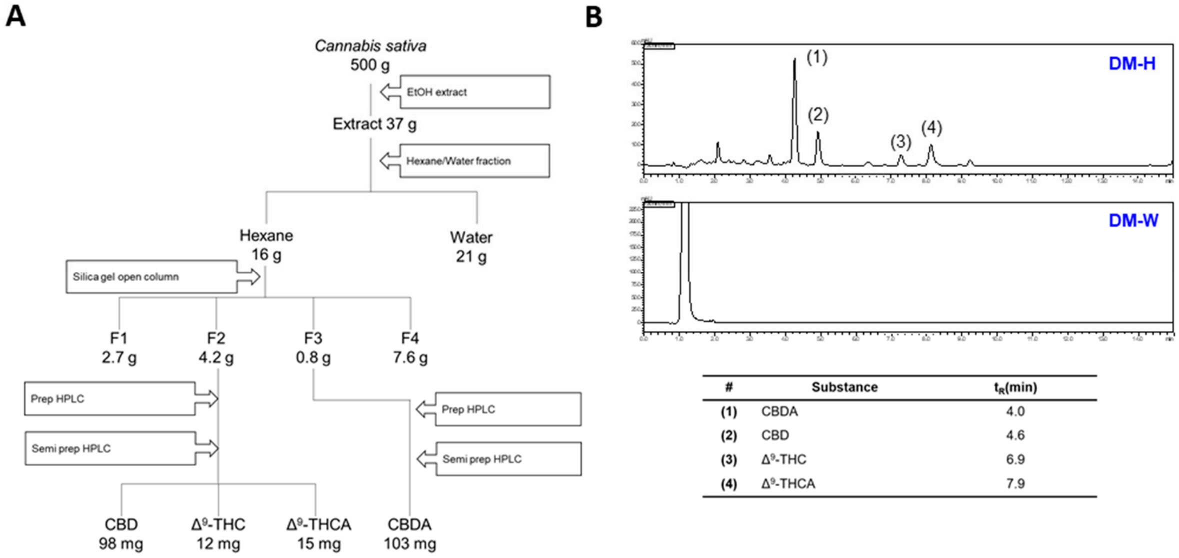

#	Substance	t_R(min)
(1)	CBDA	4.0
(2)	CBD	4.6
(3)	Δ^9-THC	6.9
(4)	Δ^9-THCA	7.9

Figure S1. Most abundant compounds of cannabis plants. (**A**) The chemical structure of cannabinoids of *Cannabis sativa*. (**B**) High-performance liquid chromatography (HPLC) of ethanolic extracts and separated cannabinoids. (B) Extraction and isolation of cannabinoids. CBDA: Cannabidiolic acid, CBD: Cannabidiol, Δ^9-THC: Tetrahydrocannabinol, Δ^9-THCA: Tetrahydrocannabinolic acid.

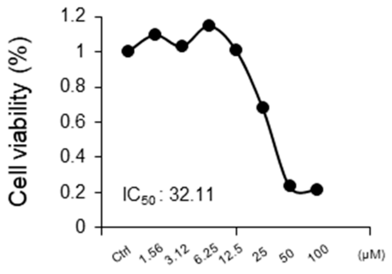

Figure S2. Cell viability of cabozantinib and CBD combination treatment in Hep3B. Cytotoxicity mediated by cabozantinib and CBD was assessed using the WST-8 assay in Hep3B.

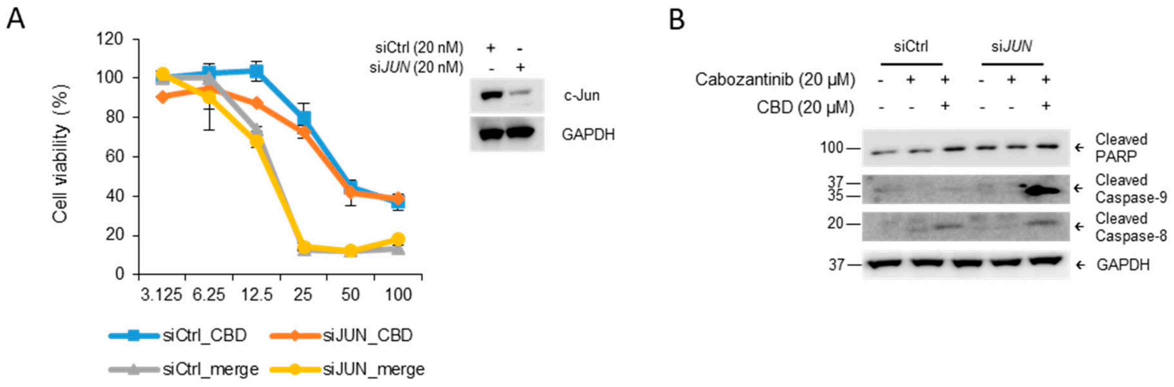

Figure S3. The effect of JUN knock-down on apoptosis after the combination treatment. (**A**) *JUN* siRNA (20 nM) and control siRNA (20 nM) were used to transfect HepG2 cells for 48 h; cytotoxicity induced by cabozantinib with and without CBD was assessed using WST-8 assay (*left*) and western blotting (*right*) at different concentrations. (**B**) *JUN* siRNA (20 nM) and control siRNA (20 nM) were used to transfect HepG2 cells, and the levels of apoptosis-related proteins (PARP, cleaved caspase-9, and cleaved caspase-8) were assessed using western blotting. Protein levels were normalized to those of GAPDH.

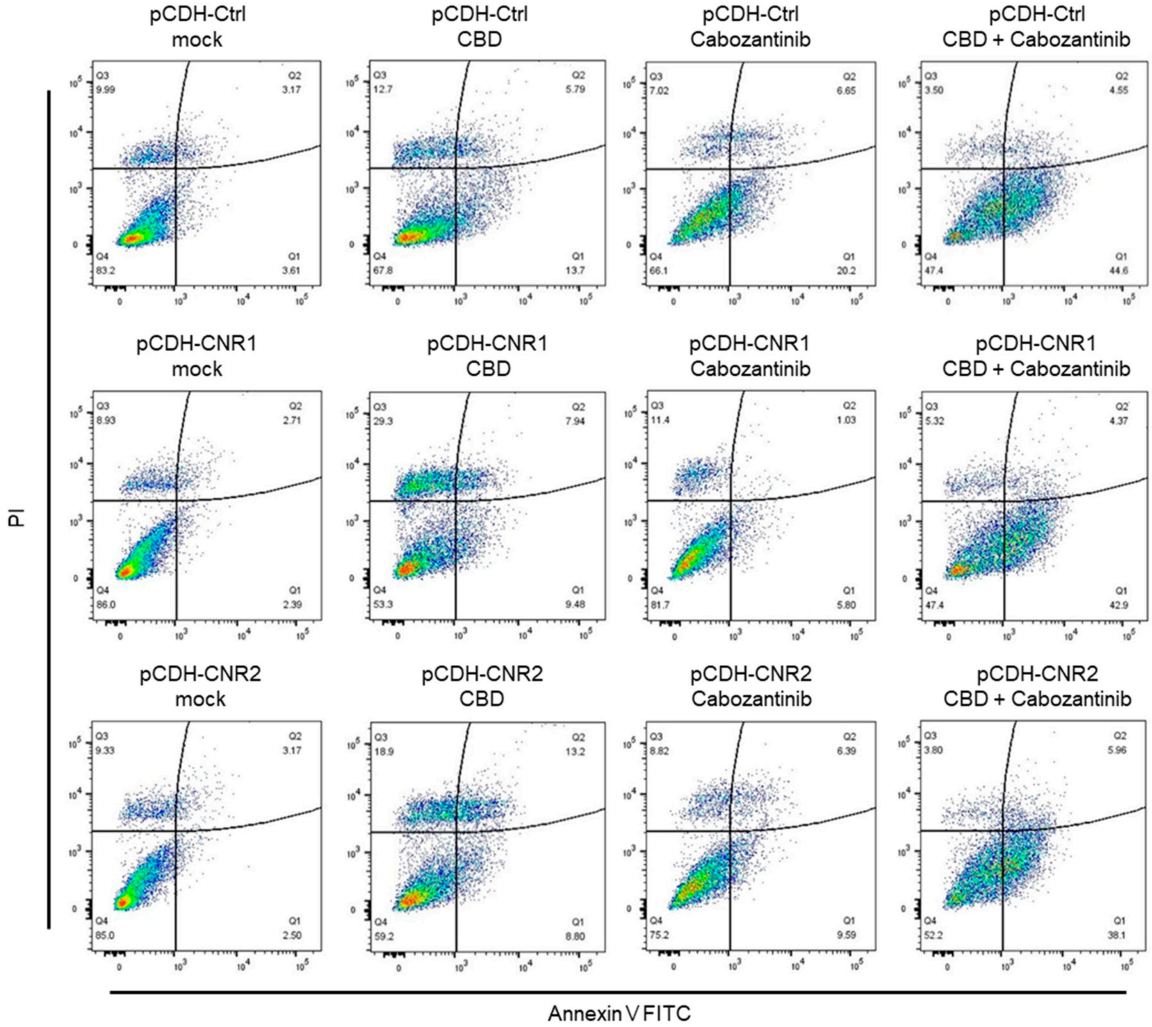

Figure S4. The effect of combination treatment in *CNR1* and *CNR2* stable cell lines. Fluorescence-activated cell sorting analysis of early apoptosis in stable cell lines incubated at the indicated condition.
